# CXC Chemokine Receptor 2 Accelerates Tubular Cell Senescence and Renal Fibrosis *via* β-Catenin-Induced Mitochondrial Dysfunction

**DOI:** 10.3389/fcell.2022.862675

**Published:** 2022-05-03

**Authors:** Ping Meng, Jiewu Huang, Xian Ling, Shan Zhou, Jingyan Wei, Mingsheng Zhu, Jinhua Miao, Weiwei Shen, Jiemei Li, Huiyun Ye, Hongxin Niu, Yunfang Zhang, Lili Zhou

**Affiliations:** ^1^ Division of Nephrology, Nanfang Hospital, National Clinical Research Center for Kidney Disease, State Key Laboratory of Organ Failure Research, Guangdong Provincial Key Laboratory of Renal Failure Research, Guangdong Provincial Institute of Nephrology, Southern Medical University, Guangzhou, China; ^2^ Department of Central Laboratory, Huadu District People’s Hospital, Southern Medical University, Guangzhou, China; ^3^ Special Medical Service Center, Zhujiang Hospital, Southern Medical University, Guangzhou, China; ^4^ Department of Nephrology, The People’s Hospital of Gaozhou, Maoming, China; ^5^ Department of Nephrology, Huadu District People’s Hospital, Southern Medical University, Guangzhou, China

**Keywords:** CXCR2, β-catenin, renal fibrosis, tubular cell senescence, mitochondrial dysfunction

## Abstract

Renal fibrosis is a common feature of various chronic kidney diseases (CKD). However, its underlying mechanism has not been totally clarified. C-X-C motif chemokine receptor (CXCR) family plays a role in renal fibrosis, however, detailed mechanisms have not been elucidated. Here, we report that CXCR2 has a potential role in tubular cell senescence and renal fibrosis, and is associated with β-catenin-activated mitochondrial dysfunction. CXCR2 is one of most increased members among CXCR family in unilateral ureteral obstruction (UUO) mice. CXCR2 was expressed primarily in tubules and co-localized with p16^INK4A^, a cellular senescence marker, and β-catenin. Administration of SB225002, a selective CXCR2 antagonist, significantly inhibited the activation of β-catenin signaling, restored mitochondrial function, protected against tubular cell senescence and renal fibrosis in unilateral ureteral obstruction (UUO) mice. In unilateral ischemia-reperfusion injury (UIRI) mice, treatment with interlukin-8 (IL-8), the ligand of CXCR2, further aggravated β-catenin activation, mitochondrial dysfunction, tubular cell senescence and renal fibrosis, whereas knockdown of p16^INK4A^ inhibited IL-8-induced these effects. *In vitro*, SB225002 inhibited mitochondrial dysfunction and tubular cell senescence. Furthermore, ICG-001, a β-catenin signaling blocker, significantly retarded CXCR2-induced cellular senescence and fibrotic changes. These results suggest that CXCR2 promotes tubular cell senescence and renal fibrosis through inducing β-catenin-activated mitochondrial dysfunction.

## Introduction

Chronic kidney disease (CKD) is characterized by continuous loss of glomerular filtration rate (GFR) (Jha et al., 2013; Cockwell and Fisher, 2020). CKD is a major cause for end-stage renal disease (ESRD) and other diseases such as cardiovascular diseases (Jha et al., 2012; Jha et al., 2013). The high morbidity and mortality of CKD brings a heavy burden to medical care and economy development (Jha et al., 2012; Nastase et al., 2018). Notably, renal fibrosis is the key pathological feature in the progression of CKD into ESRD (Gewin, 2018; Jing et al., 2020). To clarify the underlying mechanisms of renal fibrosis is valuable to retard the development of CKD and lessens the medical and financial burden.

Renal tubular epithelial cell is the key parenchymal cell type in the kidney to sustain normal kidney function. Under diseased conditions, renal tubular epithelial cells are vulnerable to the injury stimuli. They could undergo epithelial-mesenchymal transition, cell cycle arrest, and especially, cellular senescence. Reports showed that renal tubular cell senescence is an early event in renal fibrosis, and is highly associated with the progression of kidney damage (Sturmlechner et al., 2017). Cell senescence is a permanent cell cycle arrest, which is induced by DNA damage, mitochondrial dysfunction, and persistent inflammation, all of which are commonly occurred in CKD (Hernandez-Segura et al., 2018; Luo et al., 2018). With a vigorous metabolism, senescent cells can secret senescence-associated secretory phenotype (SASP) molecules such as transforming growth factor-β (TGF-β), interleukin-6 (IL-6), matrix metalloproteinases (MMPs), and interleukin-8 (IL-8). These factors could activate neighboring cells to induce and accelerate organ fibrosis, especially in kidney (Galichon et al., 2013; Luo et al., 2018; Cao et al., 2021). However, the mechanisms of cellular senescence in renal tubular cells have not been demonstrated in detail.

As a highly conserved signal, β-catenin expresses silent in adult kidney but plays a crucial role in kidney development (Wang et al., 2018; Conduit et al., 2019; Miao et al., 2019; Meng et al., 2020). However, β-catenin signaling is highly reactivated in CKD, and intimately related to cell senescence in renal tubular cells (Luo et al., 2018). One of the underlying mechanisms is dependent on β-catenin-induced mitochondrial dysfunction (Miao et al., 2019). However, the upstream inducer of β-catenin in tubular cell senescence still needs to be clarified. Our recent reports showed that β-catenin mediates the effects of G protein-coupled receptors (GPCR) (Miao et al., 2021; Zhou et al., 2021). However, the authentic role of GPCR in renal tubular cell senescence has not been elucidated, and its relationship with β-catenin is still in mystery.

The CXCR family is a large family of GPCRs, the largest superfamily of receptors with seven transmembrane domains (Kakinuma and Hwang, 2006), and belongs to chemokine receptor. They play an essential role in leukocyte migration (Kufareva et al., 2015). Recent studies showed that CXCR family is highly involved in kidney diseases (Ye et al., 2018; Yahong Liu et al., 2020; Yoo et al., 2020). In our previous study, we have found that CXCR4 promotes tubular injury and renal fibrosis through activating β-catenin (Mo et al., 2017; Yahong Liu et al., 2020). However, the role of other CXCR members, especially CXCR2, in renal fibrosis has not been thoroughly investigated.

CXCR2 is the receptor for CXCL1–3 and CXCL5–8, and plays an important role in mediating neutrophil migration (Steele et al., 2016). Previous studies showed that CXCR2 expression was increased in renal fibrosis (Wang et al., 2019), but its potential role in renal fibrosis and the underlying mechanisms have not been reported in detail. As a kind of GPCR, CXCR2 could possibly transmit signals through β-catenin. Indeed, the studies in other organs showed that CXCR2 could induce cellular senescence by activation of β-catenin (Guo et al., 2013; Zhao et al., 2017). However, the relationship between CXCR2 and β-catenin signaling and its role in renal fibrosis have not been demonstrated.

In this study, we found that CXCR2 plays a key role in renal fibrosis through inducing tubular cell senescence. CXCR2 triggered mitochondrial dysfunction and cellular senescence in tubular epithelial cells through activating β-catenin. Targeted inhibition of CXCR2/β-catenin signaling could retard renal fibrosis and retain mitochondrial homeostasis. Our findings reveal a new mechanism of renal fibrosis and implicate that targeted inhibition on CXCR2 is a new therapeutic strategy to CKD.

## Materials and Methods

### Mice and Animal Models

Six to eight-week-old C57BL/6 male mice were purchased from Southern Medical University Animal Center (Guangzhou, China) and maintained under the standard environment with normal light/dark cycle and sterile conditions. Mice were performed by UUO or UIRI surgery, as described in previous studies (Zhou et al., 2018). For UUO model, the left ureter of C57BL/6 male mice was ligated by a 4–0 silk suture in a midline abdominal incision, under general anesthesia. Mice were randomly assigned to three groups: 1) sham control, 2) UUO, 3) UUO + SB225002 (Tocris Bioscience, No. 2725). SB225002 was intraperitoneally injected at 2.5 mg/kg/d for 7 days. Mice were euthanized 7 days after UUO operation. Kidney tissues were harvested for the various analyses.

For UIRI model, C57BL/6 male mice were carried out by an established protocol (Zhou et al., 2018). Firstly, left renal pedicle was clipped by a microaneurysm clamp (item no. 18051–35; Fine Science Tools, Cambridge, United Kingdom). In the whole process of ischemia, mouse body temperature was controlled between approximately 37°C and 38°C through a metal bath system. After removing the microaneurysm clamps, reperfusion of the left ischemic kidney was visually confirmed. 10 days later, the whole right kidney was excised under sterile conditions. Mice were randomly divided into four groups: 1) sham + control-shRNA, 2) UIRI + control-shRNA, 3) UIRI + control-shRNA + IL-8 (R&D systems, 208-IL), 4) UIRI + p16^INK4A^-shRNA + IL-8. IL-8 was injected at 50 ng each mouse per day through the tail vein for 11 days. Mouse p16^INK4A^-siRNA sequence (5′-CAC​CAG​AGG​CAG​UAA​CCA​UTT-3′) was constructed on an shRNA plasmid vector (control-shRNA). The aboved mice were injected with shRNA expression plasmid (control-shRNA or p16^INK4A^-shRNA) by tail vein injection with a rapid and large volume plasmid solution as reported (Luo et al., 2018). Mice were euthanized 11 days after UIRI surgery. Serum and renal tissues were harvested for experimental analyses. All matters related to animal experiments were authorized by the Animal Ethics Committee at the Nanfang Hospital, Southern Medical University.

### Cell Culture and Treatment

Human proximal tubular epithelial cells (HKC, clone-8) were provided by Dr. Lorraine C. Racusen (Johns Hopkins University, Baltimore, MD, United States), and were cultured as described previously (Zhou et al., 2015). HKC-8 cells were stimulated with recombinant human TGF-β1 protein [(R&D Systems, Minneapolis, MN, United States) (5 ng/ml)], SB225002 [(R&D) Systems, (0.2 μM)], or ICG-001 [(847591-62-2, Chemleader, Shanghai, China) (10 μM)], respectively. Human CXCR2 siRNA sequence was 5′-GAA​CCA​GAA​UCC​CUG​GAA​ATT-3′. The transfection of siRNA into HKC-8 cells was carried out with lipofectamine 2000 (Life Technologies, Carlsbad, CA, United States). Control plasmid (pcDNA3) or CXCR2 expression plasmid (pFlag-CXCR2) was also transfected into HKC-8 cells using lipofectamine 2000 (Life Technologies, Carlsbad, CA, United States), according to instructions.

### Western Blot Assay

Protein expression was detected by western blot analysis (Zhou et al., 2018). All primary antibodies used in this study were as follows: anti-fibronectin (F3648; Sigma-Aldrich), anti-active-β-catenin (19807T; Cell Signaling Technologies), anti-PGC-1α (ab54481; Abcam), anti-p16^INK4A^ (AFS779; BD Transduction Laboratories), anti-TFAM (PB0413; BOSTER), anti-TOMM20 (ab186735; Abcam), anti-COX1 (SAB1301619; Sigma-Aldrich), anti-p19^ARF^ (SC-1665; Santa Cruz Biotechnology), anti-CPT1A (ab128568; Abcam), anti-γ-H2AX (ab26350; Abcam), anti-CXCR2 (ab14935; Abcam), anti-CXCR1 (BA0755-1; BOSTER), anti-α-tubulin (RM 2007; Ray Antibody Biotech, Beijing, China), anti-β-actin (RM 2001; Beijing Ray Antibody Biotech), anti-GAPDH (RM 2001; Ray Antibody Biotech), anti-flag-tag (KM3002; Sungene Biotech Co.).

### Reverse Transcriptase and Quantitative Real-Time Protein-Coupled Receptors

Total RNA from renal tissues and HKC-8 cells were extracted using TRIzol RNA reagent (Life Technologies, Grand Island, NY, United States). qRT-PCR experiment was carried out with an ABI PRISM 7000 instrument (Applied Bio-systems, Foster City, CA, United States). The primers used in qRT-PCR were shown in Supplementary Table S1.

### Histology and Immunohistochemical Staining

Paraffin section (6 μm) were stained using Sirius Red Staining kit (DC0040; Leagene), according to the manufacturer’s instruction. Paraffin kidney sections (3 μm) were stained with periodic acid-schiff (PAS) reagent according to the instructions. Immunohistochemical staining was performed with standard protocol. All antibodies used were as follows: anti-CXCR2 (Ab61100; Abcam), anti-p16^INK4A^ (ab189034; Abcam), rabbit anti β-catenin (ab15180; Abcam), anti-fibronectin (F3648; Sigma-Aldrich), anti-γ-H2AX (ab26350; Abcam).

### Immunofluorescence Staining

Immunofluorescence staining was performed with routine protocol (Zhou et al., 2018). Frozen kidney sections or the cover slides of cell culture were fixed with 4% paraformaldehyde at room temperature for 15 min. Primary antibodies were incubated after blocking with 10% donkey serum at room temperature for 1 h. All primary antibodies used were as follows: anti-fibronectin (F3648; Sigma-Aldrich), anti-TOMM20 (ab186735; Abcam), anti-γ-H2AX (ab26350; Abcam), anti-CXCR2 (Ab61100; Abcam), anti-β-catenin (610154; BD Transduction Laboratories), anti-Lotus Tetragonolobus Lectin (LTL) (FL-1321; VECTOR Laboratories), anti-Peanut Agglutinin (PNA) (FL-1071; VECTOR Laboratories), anti-Dolichos Biflorus Agglutinin (DBA) (FL1031; VECTOR Laboratories). Then the slides were treated with Cy3-or Cy2-conjugated secondary antibodies (Jackson Immuno-Research Laboratories, West Grove, PA, United States) for 1 h at room temperature. Nuclei were stained using DAPI (Sigma-Aldrich) under the manufacturer’s specifications.

### SA-β-Gal, MitoSOX, MitoTracker Staining

Frozen sections (3 μm) or cells cultured on coverslips were assessed by senescence β-galactosidase activity (#9860; Cell Signaling Technology), MitoTracker deep red (M22426; Thermo Fisher), mitoSOX (M36008; Thermo Fisher) staining according to the specifications.

### Transmission Electron Microscopy

HKC-8 cells were fixed in 1.25% glutaraldehyde/0.1 mol/L phosphate buffer and harvested for making ultrathin sections (60 nm). The mitochondrial morphology was viewed using an electron microscope (JEOL JEM-1010, Tokyo, Japan).

### Statistical Analyses

All data were calculated as mean ± SEM. Statistical analyses of the data were performed by SPSS 25.0 (SPSS Inc., Chicago, IL, United States). One-Way ANOVA was used for comparison between groups followed by the Least Significant Difference or Dunnett’s T3 procedure. Unpaired t test was applied to analyze the differences between two groups. Bivariate correlation analysis was performed using Pearson correlation analysis. *p* < 0.05 represents statistical significance.

## Results

### CXCR2 is Upregulated in Renal Tubular Epithelium and Associates With Tubular Senescence and Activation of β-Catenin in Chronic Kidney Diseases

**FIGURE 1:**
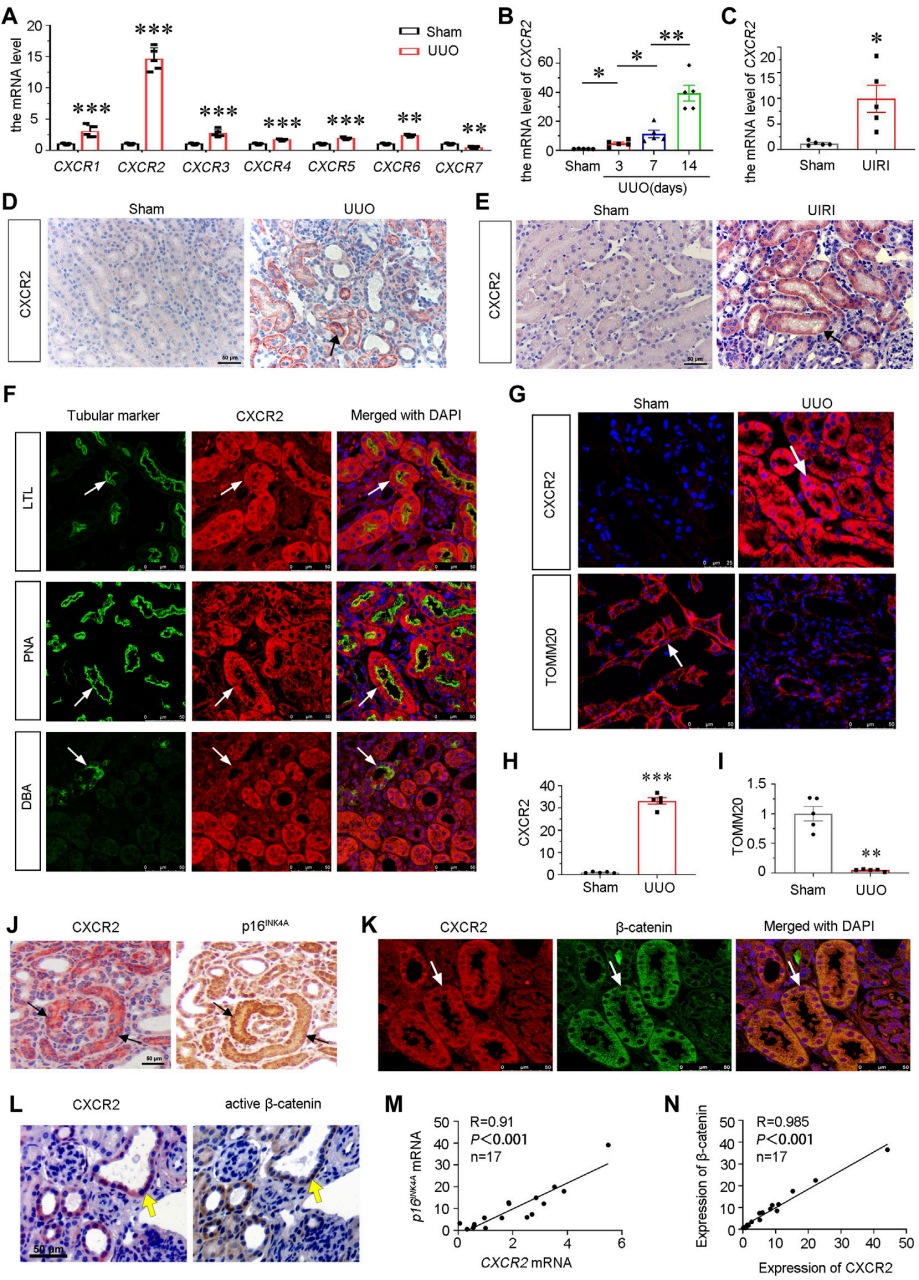
CXCR2 is upregulated in renal tubular epithelium and associated with tubular senescence and activation of β-catenin in mouse CKD model. **(A)** Graphical representations show the relative abundance of *CXCR1-7* mRNA in UUO model. ^**^ *p* < 0.01, ***p < 0.001 compare with sham controls (*n* = 5). **(B)** Graphical representations show the relative abundance of *CXCR2* mRNA level in different groups of UUO 3, 7, 14 days *p < 0.05, **p < 0.01, *n* = 5. **(C)** Graphical representations show the mRNA level of *CXCR2* in UIRI model. *p < 0.05 versus sham controls (*n* = 5). **(D,E)** Immuohistochemical staining show the expression of CXCR2 in UUO and UIRI models. Arrows indicate positive staining. Scale bar, 50 μm. **(F)** Colocalization staining of CXCR2 and various segment-specific tubular markers in the kidneys of UUO model. Frozen kidney sections were collected from the mice at 7 days after UUO. CXCR2 (red) and various segment-specific tubular markers (green) including lotus tetragonolobus lectin (LTL), peanut agglutinin (PNA), and dolichos biflorus agglutinin (DBA) were detected by immunofluorescence. Arrows indicate positive staining. Scale bar, 50 μm. **(G–I)** Representative micrographs showing the immunofluorescence staining of CXCR2 and TOMM20 in UUO model. Arrows indicate positive staining. Scale bar = 25 or 50 μm. Quantitative analysis of immunohistochemical staining for TOMM20 and CXCR2. **p < 0.01, ***p < 0.001, *n* = 5. **(J)** Colocalization of CXCR2 and p16^INK4A^ in tubules from UUO mice. CXCR2 and p16^INK4A^ were respectively immunostained using sequential paraffin-embedded kidney sections. Arrows indicate positive staining. Scale bar, 50 μm. **(K)** Colocalization of CXCR2 and β-catenin in tubules from UUO mice. Frozen renal sections from UUO 7 days mice were subjected to immunostaining of CXCR2 (red) and β-catenin (green). Arrows indicate positive staining. Scale bar, 50 μm. **(L)** Colocalization of CXCR2 and active β-catenin in tubules from UUO mice. Sequential paraffin-embedded kidney sections were immunostained for CXCR2 and active β-catenin, respectively. Arrows indicate positive staining. Scale bar, 50 μm. **(M,N)** Linear regression showing renal CXCR2 is positively correlated with **(M)** p16^INK4A^ and **(N)** β-catenin expression. The expression of renal *CXCR2* and *p16* ^*INK4A*^ mRNA levels were assessed by qRT-PCR. The correlation analysis between CXCR2 and β-catenin expressional levels was performed using immunofluorescence staining. The Pearson correlation coefficients (R) and *p* values are shown.

To identify the role of CXCR family in the pathogenesis of CKD, we first examined the expression of all members of CXCR family by qRT-PCR in mice at 7 days after UUO surgery. As shown, the mRNA levels of *CXCR1*-*6* were unregulated, while *CXCR7* was downregulated. Notably, among *CXCR* family*, CXCR2* increased most (Figure 1A), and was time-dependently upregulated in UUO mice (Figure 1B). We next assessed the expression of *CXCR*2 in other CKD models. As shown, *CXCR*2 was also increased in the kidney of UIRI mice (Figure 1C). Consistently, immunohistochemical staining showed that CXCR2 protein was also highly increased in renal tubular epithelium in UUO and UIRI mice (Figures 1D,E).

To determine the exact location of CXCR2, the co-staining of CXCR2 with specific tubular cell markers of renal different segments were performed in UUO mice. Notably, CXCR2 was largely co-localized with lotus tetragonolobus lectin (LTL), a proximal tubule marker, and peanut agglutinin (PNA), a distal tubules marker, but less co-expressed with dolichos biflorus agglutinin (DBA), a collecting duct marker (Figure 1F).

To clarify the correlation between CXCR2 and mitochondrial function, we assessed the expression of the translocase of outer mitochondrial membrane 20 (TOMM20, a mitochondrial related marker) and CXCR2 by immunofluorescence. Interestingly, as shown in Figure 1G, compared with low expression of CXCR2 in sham kidneys, CXCR2 was greatly increased in UUO-affected kidneys. On the contrary, the expression of TOMM20 showed the opposite expressional fashion, suggesting the intimate relationship between CXCR2 and mitochondrial dysfunction. Quantification analysis confirmed the upregulation of CXCR2 and the downregulation of TOMM20 in UUO (Figures 1H,I). We next assessed cellular senescence and β-catenin. As shown in Figure 1J, CXCR2 was nearly completely co-localized with tubules with positive expression of p16^INK4A^, a senescence marker. Furthermore, CXCR2 was also co-localized with β-catenin and active β-catenin in tubules (Figures 1K,L). The correlation analysis indicated that CXCR2 was positively correlated with p16^INK4A^ and β-catenin (Figures 1M,N). These results suggest that CXCR2 is highly involved in renal tubular cell senescence and mitochondrial dysfunction, and is associated with β-catenin activation.

### Blockade of CXCR2 With SB225002 ameliorates Tubular Senescence and Renal Fibrosis in Unilateral Ureteral Obstruction

**FIGURE 2:**
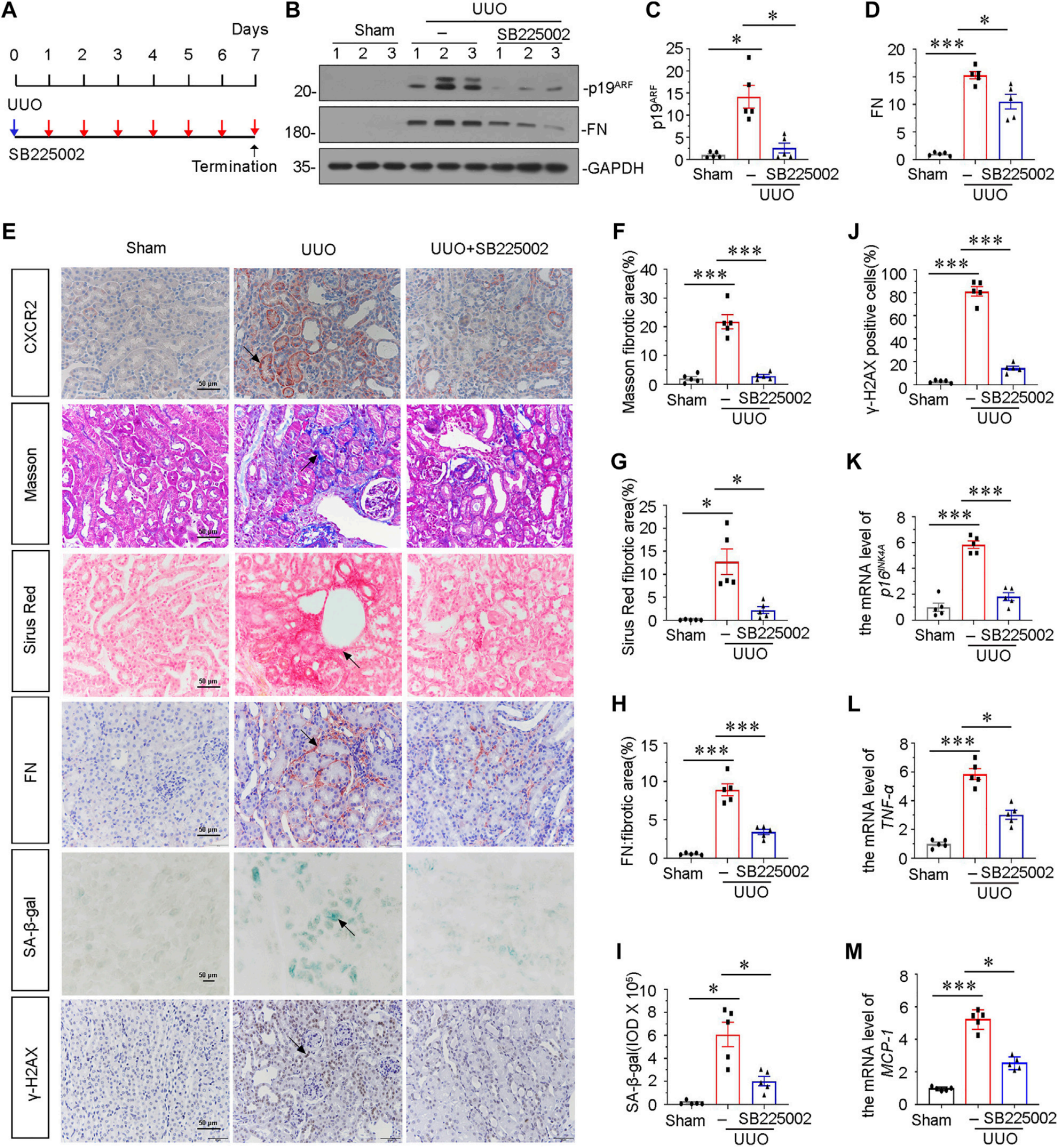
Blockade of CXCR2 with SB225002 ameliorates tubular senescence and renal fibrosis in UUO. **(A)** Experimental design. Blue arrow indicates the timing of renal UUO surgery. Red arrows indicate the injections of SB225002 (2.5 mg/kg/d). **(B)** Representative western blots showing renal expression of p19^ARF^ and fibronectin in three groups, as indicated. **(C,D)** Graphical representations of **(C)** p19^ARF^ and **(D)** fibronectin protein expressional levels are shown. *p < 0.05, ***p < 0.001, *n* = 5. **(E–J)** Representative micrographs showing Masson’s trichrome staining, Sirus Red staining, SA-β-gal staining, the fibronectin and γ-H2AX expression in each group. Frozen renal sections were stained using SA-β-gal kit. Paraffin-embedded renal sections were stained by Masson’s trichrome staining, Sirus Red staining and immunohistochemical staining. Arrows indicate positive staining. Scale bar, 50 μm. **(F–H)** Graphical representation of the extent of fibrosis which was assessed by Masson trichrome staining, Sirus Red staining and immunohistochemical staining against FN. *p < 0.05, ***p < 0.001, *n* = 5. **(I)** Quantitative graph of SA-β-gal staining in different groups, as indicated. *p < 0.05, *n* = 5. **(J)** Quantitative graph of the percentage of γ-H2AX positive cells in different groups, as indicated. ***p < 0.001, *n* = 5. **(K)** Graphical representations show the relative expression of p16^INK4A^ mRNA level in different groups. ***p < 0.001, *n* = 5. **(L,M)** Graphical representations show the relative abundance of *TNF-α* and *MCP-1* mRNA in different groups. *p < 0.05, ***p < 0.001, *n* = 5.

To explore the potential role of CXCR2 in tubular senescence, UUO mice were injected with SB225002, a specific inhibitor of CXCR2 (Figure 2A). Groups of mice were sacrificed under anesthesia at 7 days after UUO. As shown in Figures 2B–D, the protein expressional levels of p19^ARF^ and fibronectin (FN) were significantly downregulated after administration of SB225002 in UUO mice. We also examined the expression of CXCR2 in SB225002-treated mice model. As shown in Figure 2E, CXCR2 was obviously suppressed according to the immunohistochemical staining analyses. We then performed Masson trichrome and Sirus Red staining. As shown in Figures 2E–G, treatment with SB225002 significantly inhibited the progression of renal fibrosis in UUO mice. Similar results were observed when FN was stained by immunostaining (Figures 2E,H). Then we tested cellular senescence by the staining of senescence-associated β-galactosidase (SA-β-gal) activity and γ-H2AX (Figures 2E,I,J) (Luo et al., 2018; Miao et al., 2019). Quantification analysis showed that inhibition of CXCR2 by SB225002 reduced renal fibrosis and tubular senescence. Similar results were observed when the mRNA level of *p16* ^*INK4A*^ was analyzed by qRT-PCR (Figure 2K).

To examine the role of CXCR2 in inflammation, we analyzed the expression of *TNF-α* and *MCP-1* by qRT-PCR. Results showed that inhibition of CXCR2 *via* SB225002 alleviated renal inflammation (Figures 2L,M). We also assessed the expression of CXCR1. As shown in Supplementary Figures S1A,B, although CXCR1 was increased in UUO mice, the protein level of CXCR1 was not affected after SB225002 treatment.

### SB225002 Inhibits β-Catenin Signaling and Preserves Mitochondrial Function in Unilateral Ureteral Obstruction Mice

**FIGURE 3:**
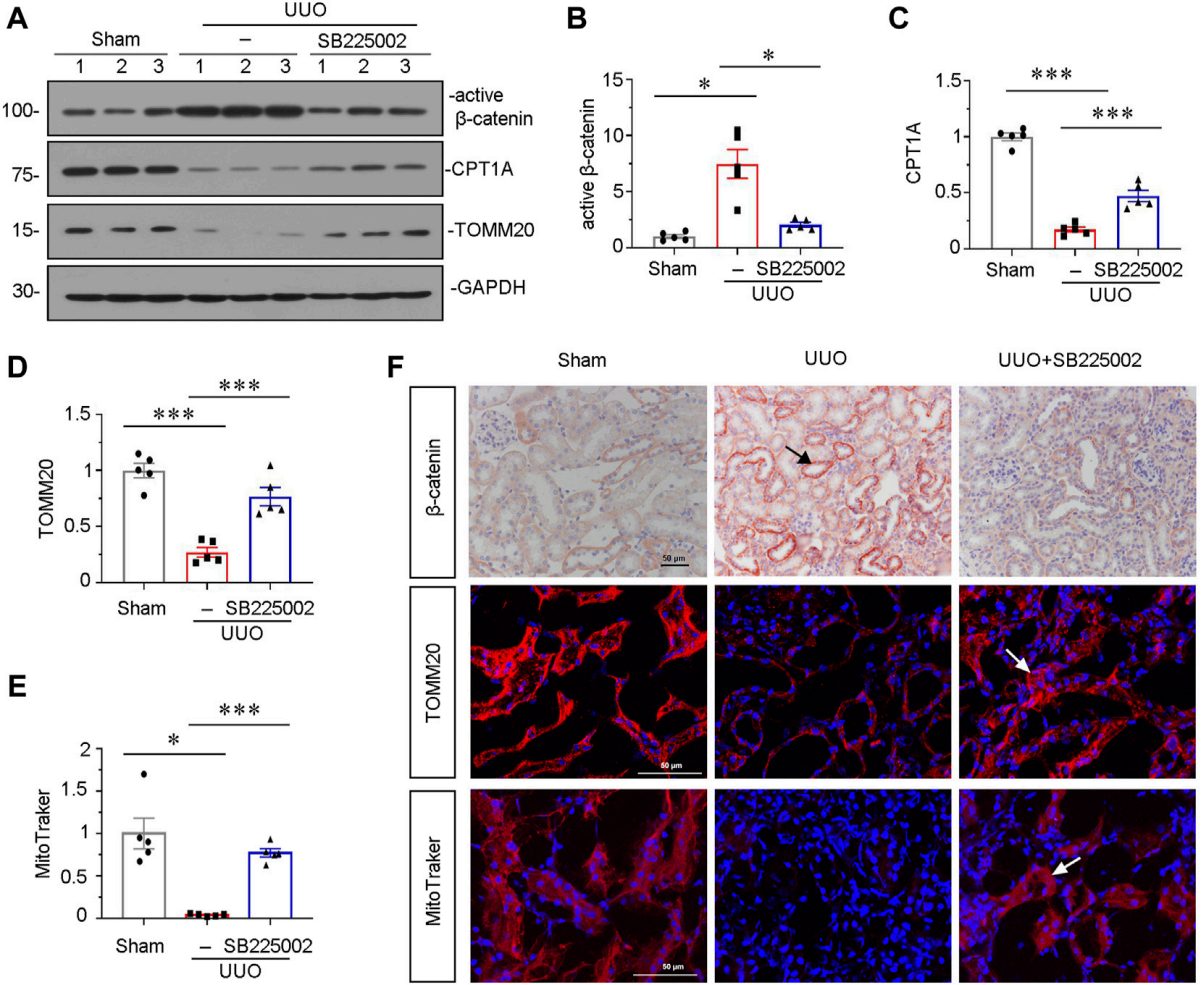
SB225002 inhibits β-catenin signaling and preserves mitochondrial functions in UUO mice model. **(A)** Protein representative graphs and expressional levels of **(B)** active β-catenin, **(C)** CPT1A, **(D)** TOMM20 are shown. *p < 0.05, ***p < 0.001 *n* = 5. **(E)** Quantitative graphs showing MitoTraker staining, which indicated mitochondrial mass in three groups. *p < 0.05, ***p < 0.001, *n* = 5. **(F)** Representative micrographs showing β-catenin, TOMM20 and MitoTraker staining in each group, as indicated. Frozen sections were stained for MitoTraker staining and TOMM20 immunofluorescence staining. Paraffin-embedded sections were stained with β-catenin antibody. Arrows indicate positive staining. Scale bar, 50 μm.

To further explore the underlying mechanisms of CXCR2 in renal fibrosis, we investigated β-catenin signaling and mitochondrial function. As shown in Figures 3A and B, administration of SB225002 significantly inhibited the expression of active β-catenin. Furthermore, the protein expressional levels of TOMM20 and the fatty acid shuttling enzyme carnitine palmitoyl-transferase 1A (CPT1A), a marker for mitochondrial function, were significantly preserved by SB225002 (Figures 3A, C,D). MitoTracker staining showed that SB225002 could inhibit the loss of mitochondrial mass (Figures 3E,F). Furthermore, consistent results were found when β-catenin and TOMM20 were tested by immunostaining (Figure 3F).

### Knockdown of p16^INK4A^ Relieves Interlukin-8-Aggravated Renal Fibrosis in Unilateral Ischemia-Reperfusion Injury Mice

**FIGURE 4:**
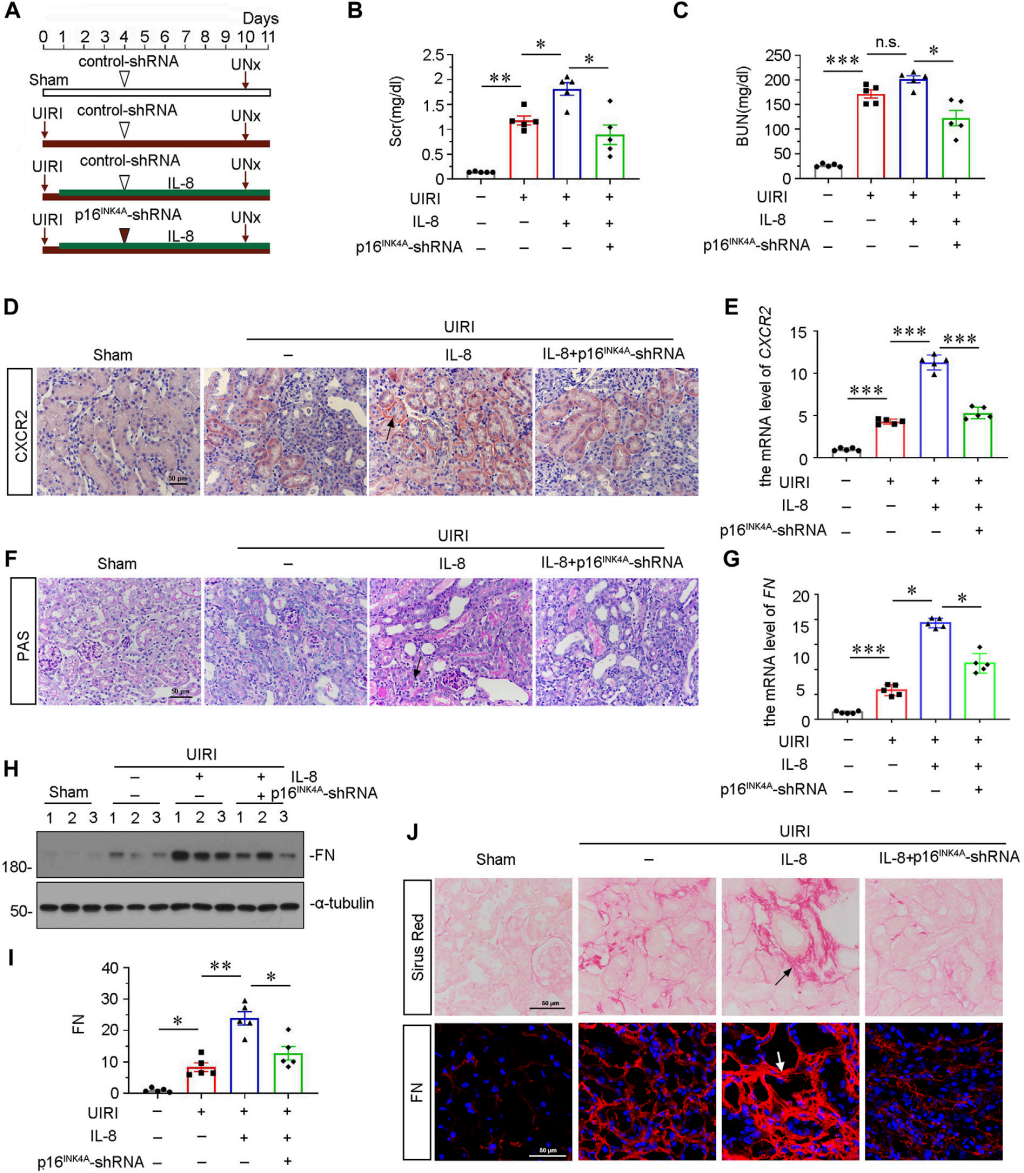
Knockdown of p16^INK4A^ inhibits IL-8-aggravated renal fibrosis in UIRI mice. **(A)** Experimental design. Triangle shows the injection of control-shRNA or p16^INK4A^-shRNA plasmid. Red arrows show the time of renal UIRI surgery and uninephrectomy. Green line segment indicates the injections of IL-8 (50 ng per time). **(B, C)** Graphic presentations show **(B)** Scr levels and **(C)** BUN levels in four groups. *p < 0.05, **p < 0.01, ***p < 0.001, *n* = 5. **(D,E)** Graphic presentations show representative micrographs of CXCR2 expression **(D)** and relative abundance of *CXCR2* mRNA **(E)** in different groups. Arrows indicate positive staining. Scale bar, 50 μm. **(F)** Representative micrographs show collagen deposition. Paraffin renal sections were subjected to periodic acid-Schiff (PAS) staining. Arrow indicates positive staining. Arrows indicate positive staining. Scale bar, 50 μm. **(G)** Graphical representations show the relative abundance of *FN* mRNA in different groups. *p < 0.05, ***p < 0.001, *n* = 5. **(H,I)** Representative western blot analysis and quantitative results show the protein expressional levels of FN in each group. *p < 0.05, **p < 0.01, *n* = 5. **(J)** Representative micrographs showing Sirus Red staining, and FN expression in four groups, as indicated. Frozen sections were stained with immunofluorescence staining for FN. Paraffin-embedded sections were stained by Sirus Red staining. Arrows indicate positive staining. Scale bar, 50 μm.

To find out a causal link between CXCR2 and tubular senescence, we assessed shRNA-mediated silencing of p16^INK4A^ in UIRI mice model injected with IL-8, a ligand of CXCR2 (Figure 4A). As shown in Figures 4B and C, serum creatinine (Scr) and blood urea nitrogen (BUN) were increased in UIRI mice, and further induced after administration of IL-8. However, these effects were suppressed by knockdown of p16^INK4A^. Furthermore, we examined the expression of CXCR2. Obviously, immumohistochemical staining and qRT-PCR results showed that CXCR2 was further activated when administration of IL-8 in UIRI mice model (Figures 4D,E). We then performed periodic acid-Schiff (PAS) staining to distinguish damaged tubules by detecting glycogen content. As shown in Figure 4F, IL-8 treatment further exacerbated tubular injury in UIRI mice, but this was inhibited by knockdown of p16^INK4A^. In addition, knockdown of p16^INK4A^ also significantly attenuated IL-8-induced upregulation of *FN* mRNA and protein expression (Figures 4G–I). Furthermore, staining of Sirus Red and immunofluorescence for FN disclosed that knockdown of p16^INK4A^ reduced renal fibrotic lesions in IL-8-treated UIRI mice (Figure 4J). As CXCR1 is also a receptor of IL-8, we examined the expression of CXCR1 in this mice model. Western blot analysis showed that CXCR1 was upregulated after UIRI surgery, however, administration of IL-8 had no effects on CXCR1 induction in UIRI mice (Supplementary Figures S1C,D).

### Knockdown of p16^INK4A^ Inhibits Interlukin-8-Induced Tubular Senescence and Mitochondrial Dysfunction in Unilateral Ischemia-Reperfusion Injury Mice

**FIGURE 5:**
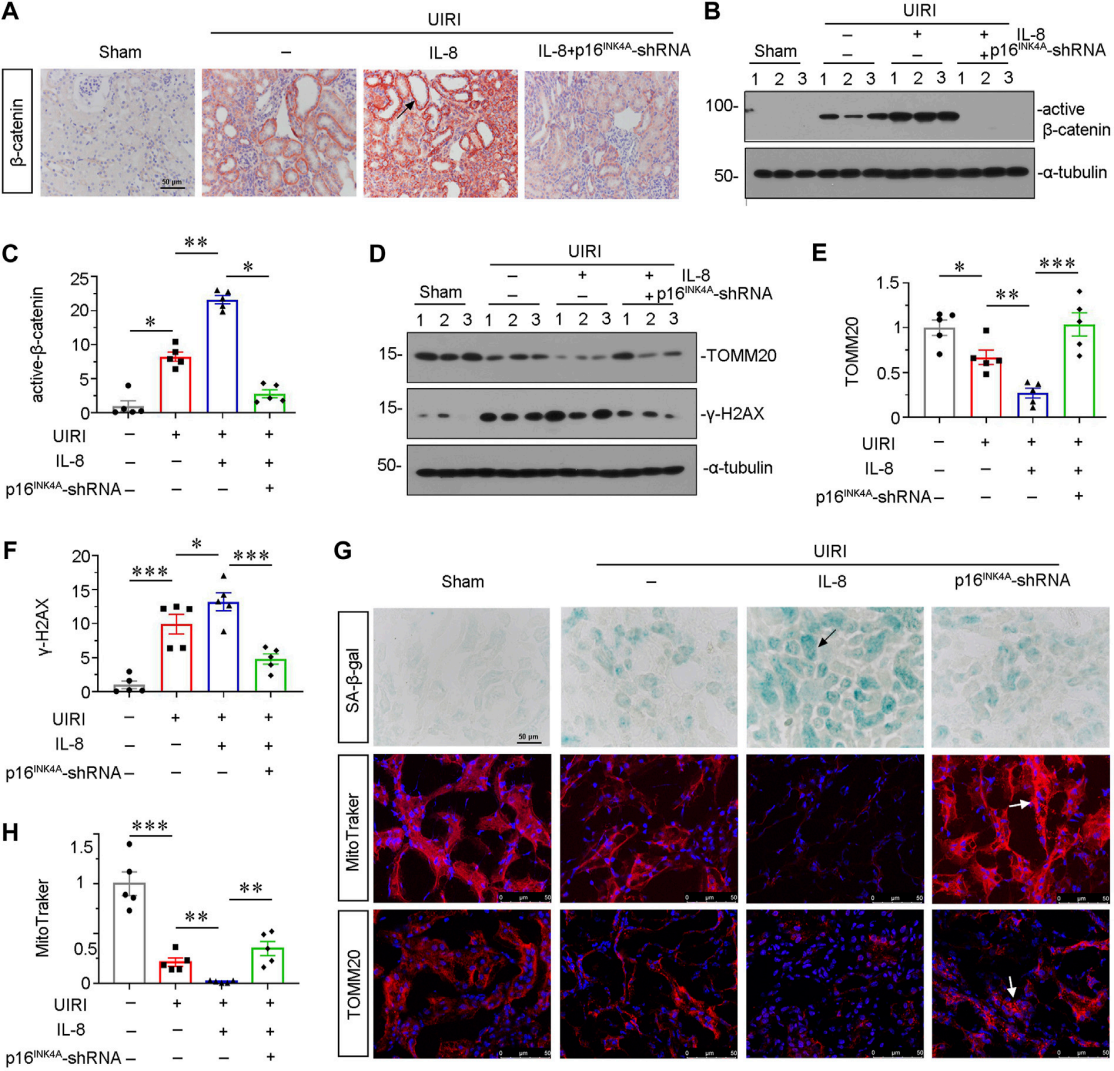
Knockdown of p16^INK4A^ inhibits IL-8-induced tubular senescence and mitochondrial dysfunction in UIRI mice. **(A)** Representative micrographs showing β-catenin expression in four groups, as indicated. Paraffin-embedded sections were stained with β-catenin antibody. Arrows indicate positive staining. Scale bar, 50 μm. **(B,C)** Representative western blot analysis and quantitative data show the protein level of active β-catenin in four groups. *p < 0.05, **p < 0.01, *n* = 5. **(D–F)** Representative western blot analysis and quantitative data show **(E)** TOMM20 and **(F)** γ-H2AX expression in different groups. *p < 0.05, **p < 0.01, ***p < 0.001, *n* = 5. **(G)** Representative micrographs showing SA-β-gal, MitoTracker and TOMM20 stainings in four groups, as indicated. Frozen sections were stained using SA-β-gal kit, and by MitoTraker staining, or were also stained with TOMM20 antibody, respectively. Arrows indicate positive staining. Arrows indicate positive staining. Scale bar, 50 μm. **(H)** Quantitative graph of MitoTraker staining showing mitochondrial mass in four groups. **p < 0.01, ***p < 0.001, *n* = 5.

We then assessed β-catenin signaling. As shown in Figures 5A–C, the immunostaining of β-catenin and western blot analyses for active β-catenin showed that IL-8 activated β-catenin singling, but inhibited by knockdown of p16^INK4A^. We then investigated the protein expression of senescent-related marker γ-H2AX and mitochondria-related marker TOMM20. As shown in Figures 5D–F, IL-8 treatment further triggered the upregulation of γ-H2AX and downregulation of TOMM20 in UIRI mice, while knockdown of p16^INK4A^ significantly inhibited these effects. Consistent results were observed when SA-β-gal activity and TOMM20 were examined by staining (Figure 5G). Furthermore, mitochondrial mass analysis by MitoTracker staining showed that IL-8 treatment significantly aggravated mitochondrial dysfunction, but blocked by knockdown of p16^INK4A^ (Figures 5G, H). These data further suggest that CXCR2 could induce tubular senescence and mitochondrial dysfunction through β-catenin activation.

### SB225002 Retards Cellular Senescence and Mitochondrial Dysfunction in Transforming Growth Factor-β1-Treated Tubular Cells *in Vitro*

**FIGURE 6:**
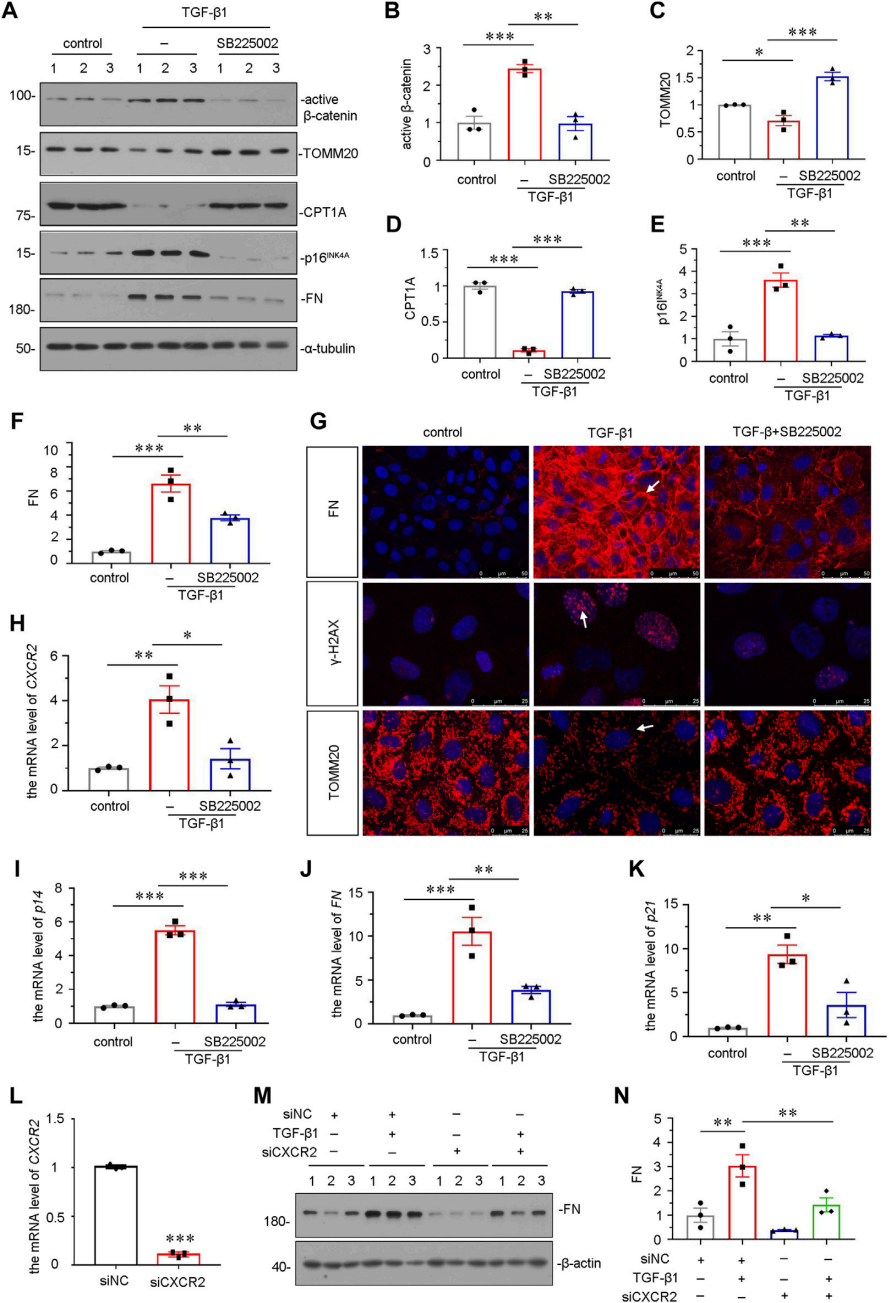
SB225002 retards cellular senescence and mitochondrial dysfunction in TGF-β1-treated tubular cells *in vitro*. **(A–F)** Representative micrographs of western blot and quantitative statistical data showing protein expressional levels of **(B)** active β-catenin, **(C)** TOMM20, **(D)** CPT1A, **(E)** p16^INK4A^ and **(F)** FN in each group. *p < 0.05, **p < 0.01, ***p < 0.001, *n* = 3. **(G)** Representative micrographs show the protein expressional level of FN, γ-H2AX and TOMM20 in each group, as indicated. Frozen sections were stained with FN, γ-H2AX and TOMM20 antibodies, respectively. Arrows indicate positive staining. Scale bar = 50 or 25 μm. **(H–K)** Graphical representations show the relative mRNA level of **(H)** *CXCR2*, **(I)** p14, **(J)** FN and **(K)** p21 in each group. *p < 0.05, **p < 0.01, ***p < 0.001, *n* = 3. **(L–N)** Graphical representations show the relative mRNA level of *CXCR2* after siRNA transfection **(L)**. ***p < 0.001, *n* = 3. Representative **(M)** Western blots and graphical representations of **(N)** FN expression in four groups. HKC-8 cells were transfected with siCXCR2 or siNC and then treated with TGF-β1 (2 ng/ml) for 24 h.

We further explored the role of CXCR2 in mediating cellular senescence and mitochondrial dysfunction *in vitro*. We adopted TGF-β1, a major fibrogenic factor, to treat human proximal tubular cell line (HKC-8) (Xi Liu et al., 2020). HKC-8 cells were pretreated with SB225002 for 1 h, then incubated with TGF-β1 for 24 h. As shown in Figures 6A and B, SB225002 could largely inhibit the upregulation of active β-catenin in TGF-β1-treated cells. We then explored mitochondrial dysfunction and cellular senescence. As shown, the protein levels of CPT1A and TOMM20 were restored by SB225002 in TGF-β1-treated HKC-8 cells (Figures 6A,C,D). Furthermore, the expressional levels of senescent-related marker p16^INK4A^ and fibrotic-related marker FN were reduced by SB225002 in TGF-β1-induced HKC-8 cells (Figures 6A,E,F). The staining also showed that SB225002 markedly reduced the expression of FN and γ-H2AX, and restored the expression of TOMM20, as shown in Figure 6G. Similar results were observed when *CXCR2*, *p14*, *FN* and *P21* mRNA levels were tested by qRT-PCR (Figures 6H–K). To further study the role of CXCR2 in renal fibrosis, we transfected HKC-8 cells with siRNA to CXCR2 or negative control (siNC). The interference efficiency of CXCR2 was firstly confirmed (Figure 6L). As shown in Figures 6M,N, interference of CXCR2 blocked the expression of FN in TGF-β1 treated HKC-8 cells.

### CXCR2 Accelerates Mitochondrial Dysfunction and Tubular Senescence *in Vitro*

**FIGURE 7:**
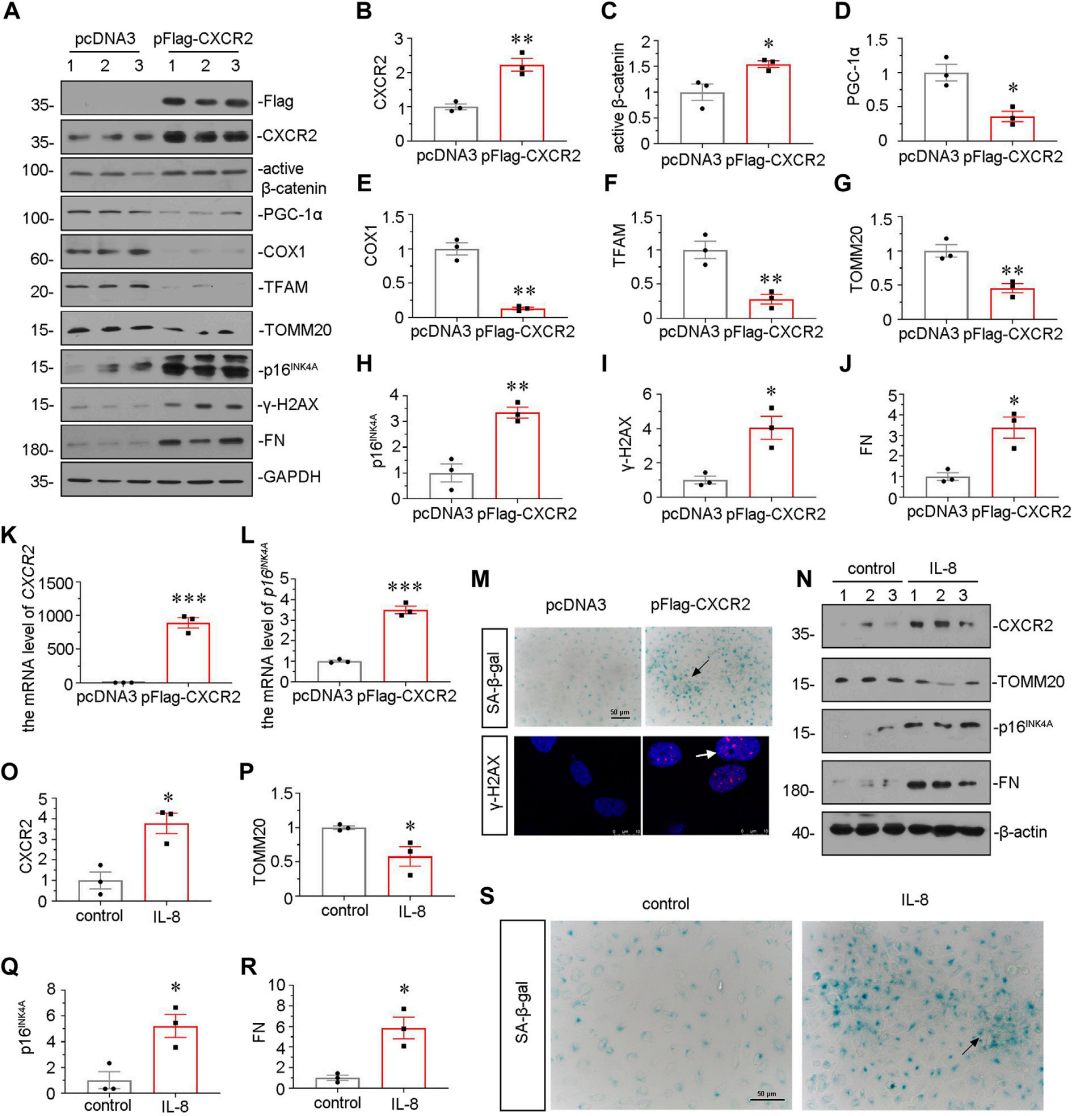
CXCR2 promotes mitochondrial dysfunction and tubular senescence *in vitro*. **(A–J)** Representative micrographs of western blot and quantitative statistical data show renal expression of **(B)** CXCR2 **(C)** active β-catenin, **(D)** PGC-1α, **(E)** COX1, **(F)** TFAM, **(G)** TOMM20, **(H)** p16^INK4A^, **(I)** γ-H2AX and **(J)** FN in each group. *p < 0.05, **p < 0.01, ***p < 0.001, versus the pcDNA3 group (*n* = 3). **(K,L)** Graphical representations show the relative mRNA level of **(K)** *CXCR2* and **(L)** *p16* ^*INK4A*^ in different groups. ***p < 0.001 versus the pcDNA3 group (*n* = 3). **(M)** Representative micrographs show SA-β-gal staining and γ-H2AX expression in different groups. Frozen sections were performed by SA-β-gal and γ-H2AX immunofluorescence staining. Arrows indicates positive staining. Scale bar = 50 or 10 μm. **(N–R)** Representative micrographs of western blot and quantitative statistical data show protein levels of **(O)** CXCR2, **(P)** TOMM20, **(Q)** p16^INK4A^ and **(R)** FN in each group. *p < 0.05 versus the control group (*n* = 3). **(S)** Representative micrographs show SA-β-gal staining in each group. Frozen renal sections were performed by SA-β-gal staining. Arrows indicate positive staining. Scale bar, 50 μm.

We further examined the effect of CXCR2 in tubular senescence and mitochondrial dysfunction *in vitro*. HKC-8 cells were transfected with CXCR2 expression plasmid. Overexpression of CXCR2 was first confirmed by western blot (Figures 7A, B). As shown in Figures 7A,C, overexpression of CXCR2 significantly promoted the upregulation of active β-catenin. Next, we examined the expressional levels of mitochondria-related proteins. As shown, the expressional levels of PGC-1α, the key transcription factor in mitochondria biogenesis, cytochrome c oxidase 1 (COX1), TFAM and TOMM20 were greatly decreased by transfection with CXCR2 expression plasmid (Figures 7A, D–G). In addition, CXCR2 overexpression aroused the expression of two senescent markers p16^INK4A^ and γ-H2AX (Figures 7A,H,I). CXCR2 also induced the upregulation of FN in HKC-8 cells (Figures 7A,J). Similar results were found when *CXCR2* and *p16* ^*INK4A*^ mRNA levels were assessed by qRT-PCR (Figures 7K,L). Moreover, as shown in Figure 7M, CXCR2 induced a significant increase in SA-β-gal activity and γ-H2AX nuclear foci in HKC-8 cells. In addition, we also test the SASP component TGF-β1 in CXCR2-overexpressed HKC-8 cells. Transfection with CXCR2 expression plasmid was significantly induced the expression of TGF-β1 (Supplementary Figure S2). These data further confirm that tubular senescence has an important role in the CXCR2-induced fibrogenic response.

HKC-8 cells were also stimulated with IL-8, a CXCR2 ligand. Treatment with recombinant protein IL-8 significantly upregulated the expression of CXCR2 (Figures 7N, O). Furthermore, IL-8 treatment downregulated the protein level of TOMM20 and upregulated the expression of p16^INK4A^ and FN (Figures 7N,P–R), suggesting the meditative role of CXCR2 in tubular senescence and mitochondrial dysfunction. IL-8 treatment also increased the activity of SA-β-gal (Figure 7S). These results suggest that CXCR2 is a strong inducer to cell senescence and mitochondrial dysfunction in renal tubular cell.

### ICG-001 Inhibits CXCR2-Aggravated Tubular Senescence and Mitochondrial Dysfunction *In Vitro*

**FIGURE 8:**
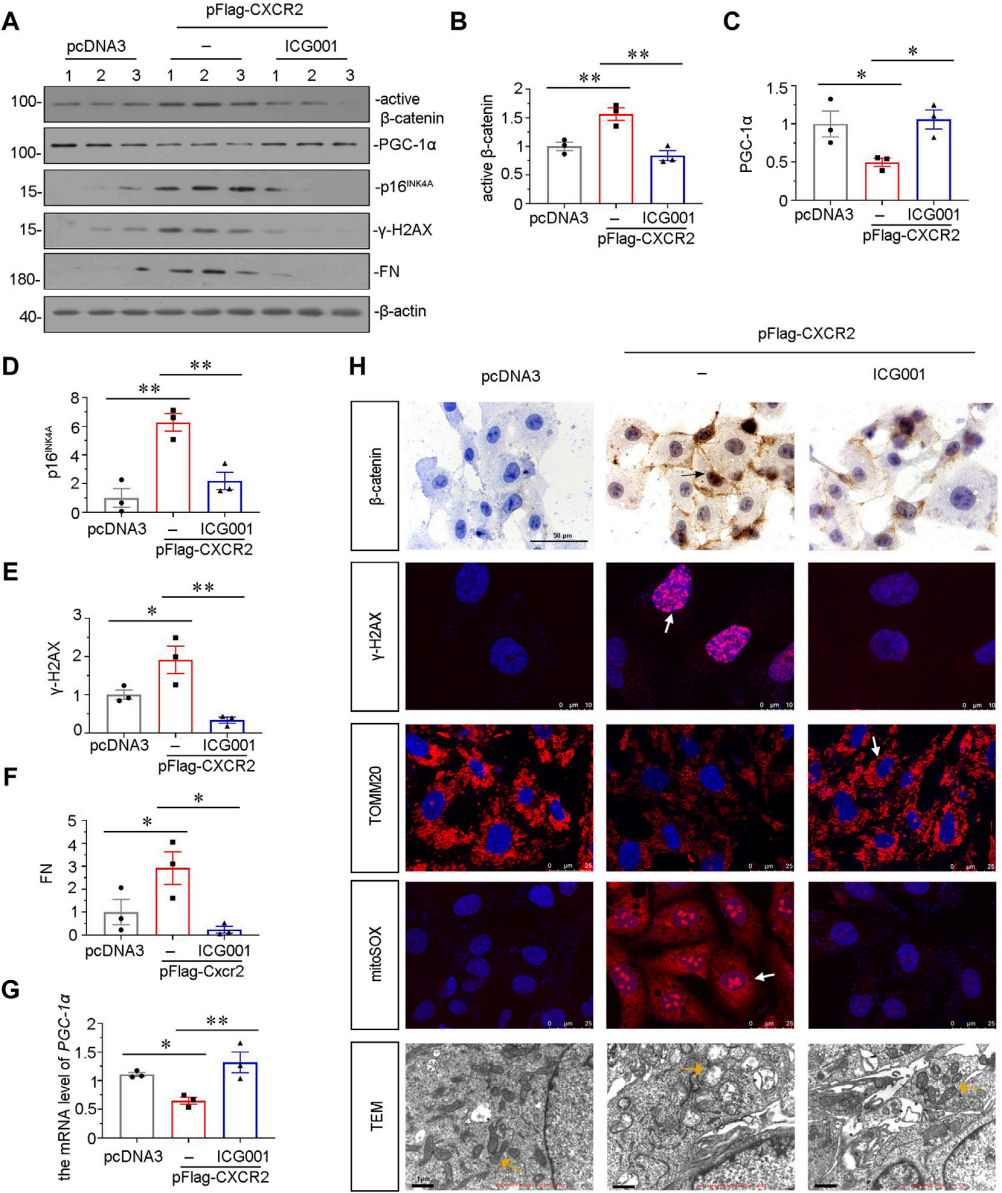
ICG-001 inhibits CXCR2-aggravated tubular senescence and mitochondrial dysfunction *in vitro*. **(A–F)** Representative micrographs of western blot and quantitative statistical data show protein levels of **(B)** active β-catenin, **(C)** PGC-1α, **(D)** p16^INK4A^, **(E)** γ-H2AX and **(F)** FN in a given group. *p < 0.05, **p < 0.01, *n* = 3. **(G)** Graphical representations show the relative abundance of *PGC-1α* mRNA in each group. *p < 0.05, **p < 0.01, *n* = 3. **(H)** Representative micrographs showing the mitochondrial ROS assessed by mitoSOX staining, and mitochondrial morphology *via* transmission electron microscopy (TEM) detection, and the expression of β-catenin, γ-H2AX and TOMM20 in different groups, as indicated. Arrows indicate positive staining. Scale bar = 50, 10, 25 or 1 μm, as indicated.

Our previous study found that β-catenin plays a central role in mitochondrial dysfunction and renal tubular senescence (Luo et al., 2018; Miao et al., 2019). To further study the downstream signals of CXCR2, we pretreated HKC-8 cells with ICG-001 (Yahong Liu et al., 2020; Zhou et al., 2021), an inhibitor of β-catenin. HKC-8 cells were pretreated with ICG-001, and then transfected with CXCR2 expression plasmid. As shown in Figures 8A–C, ICG-001 pretreatment greatly restrained CXCR2-induced increase in β-catenin and decrease in PGC-1α. In addition, the expression of p16^INK4A^ and γ-H2AX were also tested by western blot. As shown in Figures 8A,D,E, CXCR2-induced increase in p16^INK4A^ and γ-H2AX were greatly blocked by ICG-001 treatment. ICG-001 also repressed the expression of FN in CXCR2-overexpressed HKC-8 cells (Figures 8A,F). In addition, the expression of PGC-1α was also tested by qRT-PCR. As shown in Figure 8G, CXCR2-induced decrease in PGC-1α was greatly inhibited by ICG-001 treatment. Immunostaining of β-catenin showed that CXCR2 aggravated β-catenin activation in HKC-8 cells, however, treatment with ICG-001 largely inhibited it (Figure 8H). Treatment with ICG-001 markedly induced the expression of γ-H2AX, as assessed by immunofluorescence (Figure 8H). We further analyzed TOMM20, the production of mitochondrial ROS by mitoSOX staining and the ultrastructure of mitochondria using transmission electron microscopy (TEM). As shown in Figure 8H, ICG-001 greatly protected TOMM20 expression and inhibited mitochondrial ROS production in CXCR2-overexpressed tubular cells. Compared with normal mitochondria morphology in control group, CXCR2 overexpression induced the destruction of mitochondrial ultrastructure, resulting in the swelling, fragmentation and disorder of most mitochondria, while treatment with ICG-001 largely retained normal morphology of mitochondria (Figure 8H). These results further clarify that CXCR2 plays a key role in tubular senescence and mitochondrial dysfunction through activation of β-catenin.

## Discussion

With a high morbidity, mortality and heavy financial burden, CKD is becoming a public problem to the whole world (Jha et al., 2013; Minutolo et al., 2015). Renal fibrosis is a common pathological feature in all types of CKD. Notably, premature aging could be observed in the patients with normal GFR. This indicates that cellular senescence is an early event and important initiator to CKD (Miao et al., 2021). Cellular senescence is an irreversible cell cycle arrest (Calcinotto et al., 2019). Emerging evidences suggest that senescent cells could secrete SASP components of pro-inflammatory and pro-damage molecules to induce fibrosis (Ziegler et al., 2015; Cavinato et al., 2021; Yao et al., 2021). Senescent tubular cells could be easily detected in various types of CKD, and the accumulation of them is positively related with renal fibrosis (Luo et al., 2018). Our previous reports and others showed that senescent tubular epithelial cells could secret SASP molecules such as proinflammatory cytokines, growth factors, chemokines and matrix metalloproteinases, which further aggravates renal fibrosis (Liu, 2010; Luo et al., 2018). These suggest tubular cell senescence plays a fundamental role in controlling the fate of renal fibrosis. But the underlying mechanism of cellular senescence in renal tubular cells has not been clearly clarified. Therefore, an in-depth understanding of mechanisms in tubular senescence would greatly provide new clues for the treatment strategy of renal fibrosis.

Renal tubular cells are the main components of renal parenchymal cells and at a high need of energy supply to accomplish the function of the reabsorption and excretion function (Miao et al., 2021). Mitochondria are the key organelles to supply energy in the production of adenosine triphosphate (ATP) (Roger et al., 2017). Hence, the homeostasis of mitochondria is vital to tubular cell stability and its normal function. Mitochondrial dysfunction not only leads to energy absence, but also results in the production of ROS, contributing to DNA damage and cellular senescence (Di Micco et al., 2021). Our previous study showed that the activation of β-catenin was highly involved in mitochondrial dysfunction and cellular senescence, suggesting the close relationship between β-catenin and mitochondrial dysfunction (Miao et al., 2021; Zhou et al., 2021). One of the underlying mechanisms lies in the modulation of β-catenin on PGC-1α, a master controller for mitochondrial biogenesis. However, the upstream inducer to β-catenin-induced mitochondrial dysfunction and tubular cell senescence is still in mystery. Interestingly, in this study, we found that CXCR2 plays a central role in tubular cell senescence through activating β-catenin-induced mitochondrial dysfunction.

We first assessed the expression of CXCR2 in various mouse CKD models. The results indicated that CXCR2 was the most increased in CKD, among all members of CXCR family. Furthermore, CXCR2 was predominantly upregulated in tubular cells. The expression of CXCR2 had an opposite relation to TOMM20, a mitochondrial marker. We also found that CXCR2 had a positive correlation with p16^INK4A^, a senescence marker and co-localized with β-catenin, suggesting the intimate correlation with these signals (Figure 1). We also found that active β-catenin was co-localized with CXCR2. These results demonstrated that CXCR2 was highly correlated with cellular senescence and mitochondrial dysfunction through promoting β-catenin.

We next analyzed the role of CXCR2 in experimental mouse CKD models. Inhibition of CXCR2 by SB225002 significantly suppressed the expression of β-catenin and restored mitochondrial function, thereby protected against tubular senescence and renal fibrosis in UUO mice model (Figures 2, 3). Although SB225002 may have an inhibitory effect on CXCR1, we could not observe the inhibition of CXCR1 but found the inhibition on CXCR2 by SB225002 in UUO mice. This suggested that SB225002 took effects not through CXCR1. We further found that SB225002 treatment suppressed β-catenin signaling. *In vitro,* we found that siRNA-mediated inhibition of CXCR2 blocked the fibrogenesis in TGF-β1. These indicated that CXCR2 plays a crucial role in renal fibrosis. Of course, *Cxcr2* gene knockout mice could further demonstrate the role of CXCR2 in cellular senescence and renal fibrosis. This would be checked in the future study.

We also testified the association among CXCR2, β-catenin, and cellular senescence. Interestingly, the silencing of p16^INK4A^ with shRNA in UIRI mice not only reduced cellular senescence, but also inhibited the expression of β-catenin and CXCR2 (Figures 4, 5). This suggest that CXCR2, β-catenin, and cellular senescence forms a vicious cycle. As senescent cells could secret SASP molecules to further aggravate the disease progression in renal fibrosis, the targeted inhibition on each link node would certainly ameliorate the progression of the whole diseased state.

The IL-8/CXCR1/CXCR2 signaling axis is involved in the pathogenesis of several diseases (Ha et al., 2017). In this study, we found that IL-8/CXCR2 was the predominant signal for tubular cell injury and renal fibrosis. We admit IL-8 could exacerbate kidney injury besides activating CXCR2. Although CXCR1 and CXCR2 are both receptors for IL-8, we assumed that IL-8 could induce renal fibrosis mainly through activating CXCR2. As shown in Supplementary Figure S1, CXCR1 was not further elevated by IL-8 treatment in UIRI mice, but CXCR2 was strongly upregulated by IL-8 treatment. Hence, we believed that IL-8 exacerbated injury through activating CXCR2.

*In vitro*, SB225002 decreased TGF-β1-induced upregulation of active β-catenin, cellular senescence and fibrotic lesions, and restored mitochondrial function (Figure 6). Our data undoubtedly suggest that the inhibition of CXCR2 can provide a therapeutic strategy for tubular cell senescence and renal fibrosis. Although CXCR2 is not the receptor of TGF-β1, however, blocking CXCR2 could inhibit TGF-β1-induced cell senescence and mitochondrial dysfunction. We thought that CXCR2 induces cellular senescence in renal tubular cells, which could further secret SASP components such as TGF-β1, to aggravate the effects of TGF-β1 to form a vicious cycle. Hence, we could observe that the interference of CXCR2 could inhibit the effects of TGF-β1. This further suggested that CXCR2 is an essential mediator for cellular senescence in tubular cells.

The inductive role of CXCR2 in tubular cell senescence was further confirmed *in vitro.* IL-8, the ligand of CXCR2, triggered the activation of β-catenin and induced fibrotic lesions. Overexpression of CXCR2 induced β-catenin signaling activation and mitochondrial dysfunction, accelerated tubular senescence, and promoted fibrosis (Figure 7). We also confirmed the functions of ICG-001 on CXCR2-indcued effects. Interestingly, treatment of ICG-001 obviously blocked CXCR2-induced fibrosis and tubular senescence, and restored mitochondrial function (Figure 8). Overexpression of CXCR2 activated β-catenin signaling and inhibited the mitochondrial biogenesis regulator PGC-1α. All these results indicated that CXCR2 is a strong inducer to tubular cell senescence and fibrogenesis *via* the activation of β-catenin-induced mitochondria dysfunction.

Our study is at the first time to demonstrate that CXCR2 is involved in renal tubular senescence and renal fibrosis. Compared with rare expression in normal adult kidneys, the expression of CXCR2 was predominantly upregulated in tubular epithelial cells after kidney injury and associated with cell senescence. Supporting our findings, previous studies reported that CXCR2 can accelerate fibrosis in heart and lung tissues (Russo et al., 2009; Zhang et al., 2019). CXCR2 could also activate β-catenin and reinforce cellular senescence in other organs (Acosta et al., 2008; Zhao et al., 2017). Our study is at the first time to link these effects of CXCR2 in kidney area. Our findings strongly indicated that CXCR2 is an important driver for renal fibrosis *via* β-catenin-induced tubular cell senescence and mitochondrial dysfunction.

Taken together, we have shown that aberrant expression of CXCR2 plays an important role in tubular senescence and renal fibrosis. This effect is mediated by β-catenin-induced mitochondrial dysfunction. Although more studies are needed, our results provide that targeted inhibition of tubular CXCR2 serves as a new therapeutic strategy to renal fibrosis.

## Data Availability

The raw data supporting the conclusion of this article will be made available by the authors, without undue reservation.
