# Supplementary material for: CXC Chemokine Receptor 2 Accelerates Tubular Cell Senescence and Renal Fibrosis via β-Catenin-Induced Mitochondrial Dysfunction
Source: Front Cell Dev Biol. 2022 May 3;10:862675. doi: 10.3389/fcell.2022.862675 (PMC9110966; doi:10.3389/fcell.2022.862675)
Supplement: Supplementary file 1 [file DataSheet1.docx]

**Supplementary Material**

**Table S1 Primers used in qRT-PCR assay**

| Mus | *Cxcr1* | Forward 5'-3' | CAGCTGGTGCCTCAGATCAA |
| --- | --- | --- | --- |
|  |  | Reverse 5'-3' | CAGCAAGCTCAGAAGGGACA |
|  | *Cxcr2* | Forward 5'-3' | TGAGGAAGGGTGGGGAGTTC |
|  |  | Reverse 5'-3' | CGAGGTGCTAGGATTTGAGC |
|  | *Cxcr3* | Forward 5'-3' | TCAGCCAACTACGATCAGCG |
|  |  | Reverse 5'-3' | CCTCTGGAGACCAGCAGAAC |
|  | *Cxcr4* | Forward 5'-3' | GAGCATGACGGACAAGTACC |
|  |  | Reverse 5'-3' | TGGACAATAGCGAGGTACCG |
|  | *Cxcr5* | Forward 5'-3' | ACTACCCACTAACCCTGGACA |
|  |  | Reverse 5'-3' | CTGTAGGGGAATCTCCGTGC |
|  | *Cxcr6* | Forward 5'-3' | CAGGCACCTATGAGTGGGTC |
|  |  | Reverse 5'-3' | ATCTTCCACTTAGCCTGCCG |
|  | *Cxcr7* | Forward 5'-3' | GGAGCCTGCAGCGCTCACCG |
|  |  | Reverse 5'-3' | CTTAGCCTGGATATTCACCC |
|  | *Fn* | Forward 5'-3' | GATGAGCTTCCCCAACTGGT |
|  |  | Reverse 5'-3' | CTGGGTTGTTGGTGGGATGT |
|  | *β--actin* | Forward 5'-3' | CAGCTGAGAGGGAAATCGTG |
|  |  | Reverse 5'-3' | CGTTGCCAATAGTGATGACC |
|  | *TNF-α* | Forward 5'-3' | GACCCTCACACTCAGATCA |
|  |  | Reverse 5'-3' | ACTTGGTGGTTTGCTACG |
|  | *MCP-1* | Forward 5'-3' | tggggacaccttttagcatc |
|  |  | Reverse 5'-3' | cttgaaggtgttgccctc |
|  | *P16^INK4A^* | Forward 5'-3' | CTGGGTGCTCTTTGTGTT |
|  |  | Reverse 5'-3' | GTGCTTGAGCTGAAGCTATG |
|  | *TGF-β1* | Forward 5'-3' | GTGGAAATCAACGGGATCAG |
|  |  | Reverse 5'-3' | GTTGGTATCCAGGGCTCTCC |

| Human | *CXCR2* | Forward 5'-3' | CATGGCTTGATCAGCAAGGA |
| --- | --- | --- | --- |
|  |  | Reverse 5'-3' | TGGAAGTGTGCCCTGAAGAAG |
|  | *PGC-1α* | Forward 5'-3' | TGTGCAACTCTCTGGAACTG |
|  |  | Reverse 5'-3' | TGAGGACTTGCTGAGTGGTG |
|  | *FN* | Forward 5'-3' | TTGCAGTGAGCCATGGGAG |
|  |  | Reverse 5'-3' | TCCTGTTATCTGGGCCCGA |
|  | *P14* | Forward 5'-3' | CTGATCTCCATCGCAGGGAC |
|  |  | Reverse 5'-3' | ATGTCCACGAGGTCCTGAGC |
|  | *P21* | Forward 5'-3' | GTCACTGTCTTGTACCCTTGTG |
|  |  | Reverse 5'-3' | CGGCGTTTGGAGTGGTAGAAA |
|  | *P16^INK4A^* | Forward 5'-3' | ACTTCAGGGGTGCCACATTC |
|  |  | Reverse 5'-3' | CGACCCTGTCCCTCAAATCC |
|  | *GAPDH* | Forward 5'-3' | GCACCGTCAAGGCTGAGAAC |
|  |  | Reverse 5'-3' | TGGTGAAGACGCCAGTGGA |

**
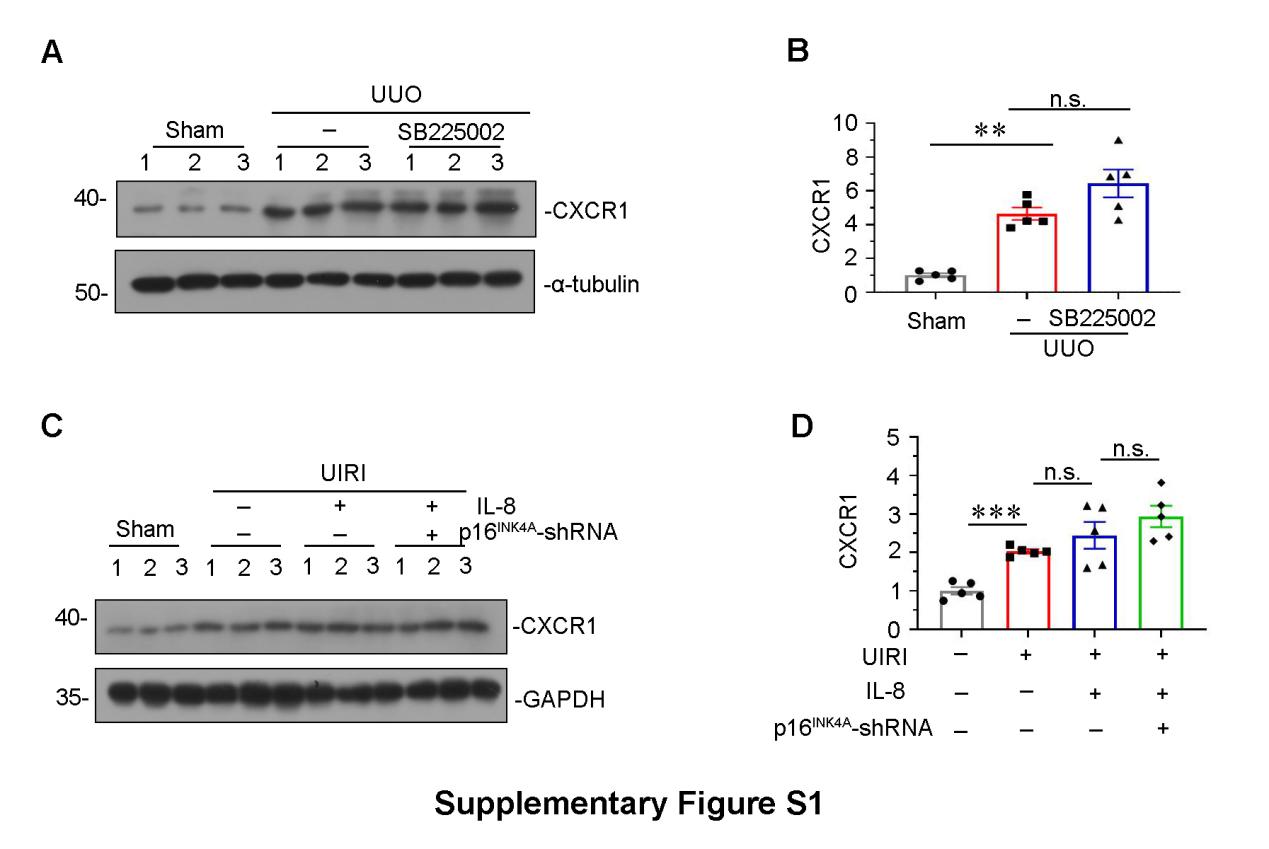
**

**Supplementary Figure S1**

1. **B**) Representative western blot and quantitative data show renal expression of CXCR1 in a given group. ***P <* 0.01, n = 5. (**C-D**) Representative western blot and quantitative data show renal expression of CXCR1 in a given group. ****P* *<* 0.001, n = 5.


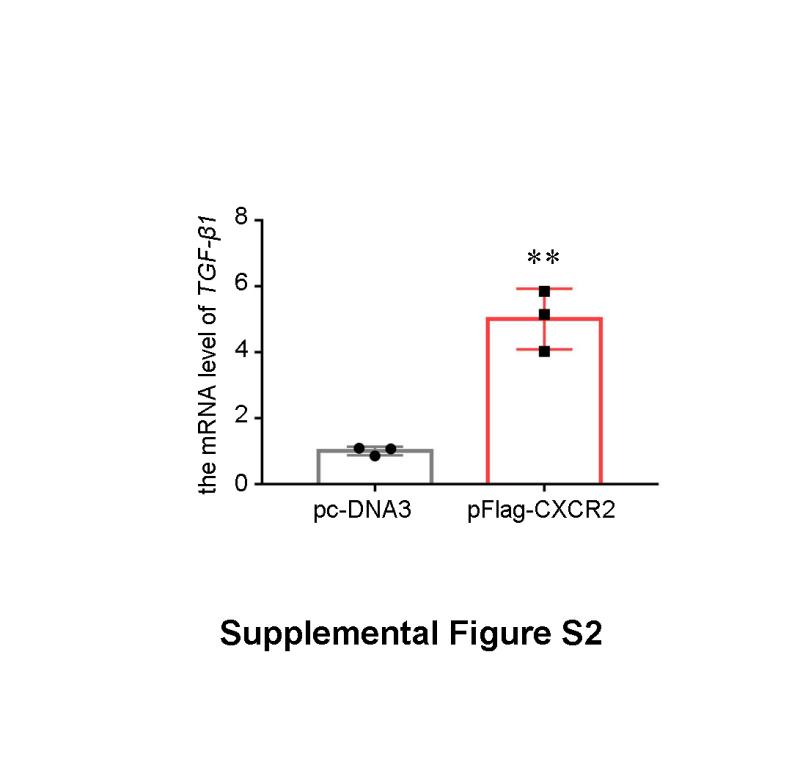


**Supplementary Figure S2**

Representative qRT-PCR data show the mRNA level of *TGF-β1* in pFlag-CXCR2 group versus the pcDNA3 group. ***P <* 0.01, n = 3.
